# A Survey of Canadian Psychiatrists’ Attitudes Towards Palliative Care Approaches for Severe and Persistent Mental Illness: Enquête sur les attitudes des psychiatres canadiens à l’égard des approches de soins palliatifs pour les troubles mentaux graves et persistants

**DOI:** 10.1177/07067437261474291

**Published:** 2026-08-12

**Authors:** Sarah Levitt, Lucy Panko, Kate Tsiandoulas, Micaela Forte, Lucy Costa, Daniel Z. Buchman

**Affiliations:** 1 Mental Health Program, 7989 University Health Network, Toronto, Ontario, Canada; 2 Department of Psychiatry, Temerty Faculty of Medicine, 7938 University of Toronto, Toronto, Ontario, Canada; 3 7978 Centre for Addiction and Mental Health, Toronto, Ontario, Canada; 4 206712 Institute of Health Policy, Management and Evaluation, Toronto, Ontario, Canada; 5 Dalla Lana School of Public Health, 7938 University of Toronto, Toronto, Ontario, Canada; 6 Joint Centre for Bioethics, 7938 University of Toronto, Toronto, Ontario, Canada; 7 Empowerment Council, 7978 Centre for Addiction and Mental Health, Toronto, Ontario, Canada

**Keywords:** palliative psychiatry, severe and persistent mental illness, futility, psychiatrists, anorexia nervosa, schizophrenia, major depressive disorder

## Abstract

**Background:**

Palliative psychiatry, which emphasizes improving quality of life and reducing suffering for individuals with severe and persistent mental illness (SPMI), has gained recognition internationally. Canadian psychiatrists’ perspectives on palliative psychiatry remain under-explored. We adapted a survey created by Trachsel et al. on the acceptability of palliative psychiatry amongst German-speaking Swiss psychiatrists for the Canadian context to address this gap.

**Method:**

We conducted a cross-sectional survey of Canadian psychiatrists (*n* = 69). We collected data on demographics, attitudes towards palliative care in psychiatry, and responses to 3 case vignettes describing a patient experiencing anorexia nervosa, schizophrenia, or major depressive disorder, respectively. Additional questions explored participants’ views on coercion. Participants could elaborate on their responses to the case vignettes using optional free-text boxes. We analyzed the survey data using descriptive statistics and the qualitative data using structured tabular thematic analysis.

**Results:**

Participants broadly endorsed treatment goals focused on improving function, reducing suffering, preventing suicide, and maintaining patient autonomy, while placing less emphasis on curative approaches. Participants were divided on whether palliative care relates to end-of-life care. There was strong acceptance for a palliative approach in SPMI care. Attitudes towards prognosis and treatment futility varied by diagnosis amongst the case vignettes. Participants expressed nuanced views on coercion, highlighting ethical tensions when considering interventions contrary to patient preferences. Our qualitative analysis uncovered themes of ethical dimensions, clinical paradigms, characteristics of mental illness, considering comprehensive treatment options, psychosocial factors, and systemic factors in participants’ consideration of their responses.

**Conclusions:**

This cohort of Canadian psychiatrists largely endorses the applicability of palliative care approaches within SPMI care. Our findings underscore the need for further research with larger samples to understand potential gaps between theoretical acceptance and practical implementation. Future research should include the perspectives of patients, families, and other interest-holders to guide the potential integration of a palliative care model into psychiatric practice.

## Plain Language Summary

A Survey of Canadian Psychiatrists Found That Participants Largely Agree That Palliative Care Approaches Can Be Applicable in Caring for People Experiencing Severe and Persistent Mental Illness.

Plain Language Summary

Palliative psychiatry is an emerging possible type of care for individuals experiencing severe and persistent mental illness (SPMI). This approach focuses on interventions that improve quality of life (QOL) and/or reduce suffering, rather than necessarily trying to cure illness or eliminate symptoms. Examples of SPMI include anorexia nervosa, schizophrenia, and major depressive disorder. Palliative psychiatry is a new way of thinking about mental healthcare. It remains underexplored what interest-holders, such as Canadian psychiatrists, think about it. In 2019, researchers used a survey to ask German-speaking Swiss psychiatrists about their opinions and attitudes towards palliative psychiatry and other related topics using general questions and case examples. This study replicates that survey with 69 Canadian psychiatrists. The group of Canadian psychiatrists surveyed broadly agreed that palliative care principles and approaches could benefit their patients, and that there are instances of SPMI where prioritizing QOL over longevity or cure seems important. This study also analyzed the comments participants left on the survey when answering the questions. Psychiatrists’ comments revealed complicated decision-making when considering questions of palliation for their patients, involving thinking about ethics, psychological factors, social circumstances, healthcare systems, and possible treatment options and approaches. We could not draw any broad conclusions about what all Canadian psychiatrists think from this survey. These results suggest future research (with a bigger group) is needed to understand how health systems can adapt palliative care approaches within mental healthcare and should also explore what other interest-holders (such as people living with mental illness and their support persons) think about integrating palliative approaches into mental healthcare.

## Résumé

**Contexte:**

La psychiatrie palliative, qui met l’accent sur l’amélioration de la qualité de vie et la réduction de la souffrance des personnes atteintes de troubles mentaux graves et persistants, est désormais reconnue à l’échelle internationale. Le point de vue des psychiatres canadiens sur la psychiatrie palliative reste toutefois peu étudié. Afin de combler cette lacune, nous avons adapté au contexte canadien une enquête élaborée par Trachsel et al. sur l’acceptabilité de la psychiatrie palliative auprès des psychiatres suisses germanophones.

**Méthodes:**

Nous avons mené une enquête transversale auprès de psychiatres canadiens (n = 69). Nous avons recueilli des données démographiques, évalué les attitudes à l’égard des soins palliatifs en psychiatrie et analysé les réponses à trois études de cas cliniques décrivant respectivement un patient souffrant d’anorexie mentale, de schizophrénie et de trouble dépressif majeur. D’autres questions ont permis d’étudier le point de vue des participants sur la contrainte. Les participants pouvaient préciser leurs réponses aux études de cas à l’aide de zones de texte libre facultatives. Nous avons analysé les données de l’enquête à l’aide de statistiques descriptives et les données qualitatives à l’aide d’une analyse thématique tabulaire structurée.

**Résultats:**

Les participants ont largement approuvé les objectifs de traitement axés sur l’amélioration des capacités fonctionnelles, la réduction de la souffrance, la prévention du suicide et le maintien de l’autonomie du patient, tout en accordant moins d’importance aux approches curatives. Les participants étaient divisés sur la question de savoir si les soins palliatifs étaient liés aux soins de fin de vie. L’adoption d’une approche palliative dans la prise en charge des troubles mentaux graves et persistants a été largement acceptée. Les attitudes à l’égard du pronostic et de la futilité du traitement variaient selon le diagnostic présenté dans les études de cas cliniques. Les participants ont exprimé des points de vue nuancés sur la contrainte, en soulignant les tensions éthiques lorsqu’ils envisagent des interventions contraires aux préférences des patients. Notre analyse qualitative a mis en évidence des thèmes comme les dimensions éthiques, les paradigmes cliniques, les caractéristiques de la maladie mentale, la prise en compte d’options thérapeutiques globales, les facteurs psychosociaux et les facteurs systémiques dans l’examen des réponses des participants.

**Conclusions:**

Cette cohorte de psychiatres canadiens est largement en faveur des approches de soins palliatifs dans le cadre de la prise en charge des troubles mentaux graves et persistants. Nos résultats soulignent la nécessité de mener des recherches supplémentaires sur des échantillons plus volumineux pour comprendre les écarts potentiels entre l’acceptation théorique et la mise en œuvre pratique. Les recherches futures devraient inclure le point de vue des patients, des familles et d’autres parties prenantes afin d’orienter l’intégration potentielle d’un modèle de soins palliatifs dans la pratique psychiatrique.

## Introduction

Severe and persistent mental illness (SPMI) refers to experiences of mental illness of prolonged symptom duration (e.g., treatment-resistant bipolar disorder, schizophrenia, or anorexia nervosa (AN)) that lead to significant impairment.^1^ Those affected are at risk of both therapeutic neglect and overly aggressive interventions^2^ associated with mental healthcare-as-usual, which prioritizes maximal symptom control.^3^ Recovery-oriented care improves support for individuals experiencing SPMI by focusing on meaning-making in life. It builds resilience by promoting a sense of mastery over one's condition and encouraging participation in society alongside symptom mitigation.^4^ More recently, palliative psychiatry was introduced as an alternate approach to mental healthcare that aims to improve the quality-of-life (QOL) for individuals experiencing SPMI by reducing the risk of iatrogenic harms associated with potentially futile interventions and minimizing suffering.^5^ Palliative psychiatry might work around, rather than attempt to eliminate, symptoms and accepts that some individuals experiencing SPMI sustain symptoms that cannot be modified despite best efforts.^3,6^ Palliative psychiatry contends that patients should not be abandoned, but rather provided life-affirming care that prioritizes patient preferences for QOL.^7^

There is no singular approach or care pathway for palliative psychiatry.^7^ Critics argue that palliative psychiatry may complicate access to high-quality care for persons experiencing SPMI,^8^ worsen stigma and distress associated with the word “palliative,”^9^ and will inevitably lead to Medical Assistance in Dying with Mental Illness as the Sole Underlying Medical Condition (MAiD-MI-SUMC).^10^ Some authors question the ability to ethically palliate mental illness given the diagnostic instability resulting from a lack of biomarkers, prognostic uncertainty, and concern that this approach may lead to therapeutic nihilism.^11^ However, an early palliative psychiatry care model from Belgium, Oyster Care, has demonstrated benefits to individuals experiencing SPMI, such as reduced aggression and self-mutilation, and fewer transfers to acute care in the Belgian context.^12^ Nascent evidence suggests that palliative psychiatry may be acceptable to interprofessional mental health clinicians across healthcare systems and cultures. Surveys of both German-speaking Swiss and Indian psychiatrists indicated an acceptability for palliative approaches in SPMI care.^13,14^ A Swiss pilot study found most psychiatric nurses agreed that palliative care was indicated in certain instances of SPMI.^15^

Given the differences across mental healthcare legislation, resource availability, and cultural attitudes towards mental illness, it is difficult to ascertain the acceptability of palliative psychiatry for Canadian psychiatrists from the existing studies. Accordingly, Canadian psychiatrists’ attitudes towards palliative psychiatry remain unknown. Recent discussions in the academic literature highlight that psychiatrists have diverse perspectives on concepts adjacent to palliative psychiatry, such as irremediability, treatment futility, psychiatric suffering, and support/abandonment when providing mental healthcare to individuals experiencing SPMI.^11,16–18^ Moreover, palliative psychiatry approaches have been proposed elsewhere as a potential alternative to MAiD-MI-SUMC,^19,20^ an intervention whose ethical legitimacy has been debated intensely by Canadian psychiatrists in recent years.^21,22^ It is thus timely to understand how Canadian psychiatrists specifically are reflecting on palliative psychiatry. Our study examines Canadian psychiatrists’ attitudes towards palliative psychiatry (and related concepts such as terminal mental illness and psychiatric futility) to address this knowledge gap and to inform broader discussions on its acceptability across health systems and cultural contexts.

## Method

### Study Design

This multi-methods study adapts Trachsel et al.'s 2019 survey^13^ on palliative psychiatry for a cross-sectional survey of Canadian psychiatrists (see Appendix 1). The survey gathered demographic information and Likert-scale responses on agreeability with statements about palliative care and related concepts within mental healthcare. Participants received the WHO definition of palliative care but otherwise interpreted the questions independently, reflecting the lack of consensus definitions for concepts such as futility in mental healthcare. The survey then presented 3 case vignettes, each describing a different instance of someone experiencing SPMI. These vignettes were adapted from the original survey to reflect Canadian practice and the language of Canadian mental health legislation. The adapted vignettes were reviewed by Canadian experts prior to their inclusion in the survey. We gathered Likert-scale responses on agreeability about the applicability of palliative psychiatry and related concepts within each case vignette. Participants could provide optional free-text responses to elaborate qualitatively on their responses. This study was approved by the Centre for Addiction and Mental Health Research Ethics Board 2022/246.

### Recruitment and Sample

We recruited psychiatrists who were 18 years or older with an independent licence to practice psychiatry, a primary practice in Canada, and who spoke English or French. Participants were recruited through the professional networks of the research team, the Canadian Psychiatric Association newsletter, the Association des Medecins Psychiatres Du Quebec, and social media (e.g., closed Facebook groups with psychiatrist members, and LinkedIn posts). We distributed the survey using digital posters that contained a contextual introduction and a QR code to access the questions online. Digital posters were distributed in both English and French. The survey and its associated consent forms were translated from English to French professionally, and available in both languages. Our recruitment strategy was designed to reach as many psychiatrists as possible to capture diverse opinions and mitigate potential bias.

The English survey was available between 23 March 2023 and 31 March 2025, and the French survey was available between 5 August 2023 and 31 March 2025. No survey responses were collected between 31 August 23 and 09 January 2025. No honorarium was provided for survey completion.

### Data Collection

We distributed the survey digitally, hosted on REDCap. Participants provided written informed consent through a checkbox on the landing page before accessing the survey. We collected demographic information. The survey used a 5-point Likert scale (1 = strongly disagree to 5 = strongly agree) to gauge participants’ level of agreement with statements related to the goals of psychiatric care. After reviewing the WHO definition of palliative care,^23^ participants used the same 5-point Likert scale to answer questions about (1) the applicability of palliation and related concepts to mental healthcare, and (2) the applicability of palliative psychiatry and related concepts to 3 case vignettes describing fictionalized narratives of symptoms and treatment courses of different instances of SPMI (AN, schizophrenia, and major depressive disorder (MDD)). Optional free-text boxes allowed participants to elaborate on their case vignette responses. Once data collection was completed, all free-text responses written in French were translated into English by a bilingual research analyst with translation experience who mitigated the risk of semantic drift by using dictionaries and accessing academic French materials discussing similar concepts.

### Data Analysis

We analyzed the quantitative data using descriptive statistics. We used a single post-hoc analysis to test a hypothesis that emerged from the initial data analysis. We used Structured Tabular Thematic Analysis (ST-TA) to analyze free-text responses in Microsoft Excel, following an inductive approach.^24^ Two team members (LP, KT) transcribed and coded the data independently, reflecting on their own assumptions, personal and disciplinary backgrounds and potential biases throughout, which the research team reviewed to agree on themes and subthemes. The coding process was iterative and occurred over several meetings with the larger research team. We sought inter-analyst (LP, KT) consensus through discussion of the texts and codes. Disagreements were resolved through collaborative discussions with the wider research team, and coding decisions were documented for transparency. We calculated theme and subtheme frequencies, noting that subtheme frequency could exceed 100% as multiple themes or subthemes could be applied to a single free-text response.

## Results

### Participant Demographics

Sixty-nine participants completed the survey (*n* = 52 in English, *n* = 17 in French). 69.6% (*n* = 48) of participants were women. Participants’ ages ranged between 35 and 75+ years old, with the highest concentration of participants identifying as 35–44 years old (40.6%, *n* = 28). Participants were geographically distributed across British Columbia, Alberta, Manitoba, Ontario, Quebec, and Newfoundland and Labrador. Participants identified across diverse ethnic groups, including white European, white North America, Middle Eastern, Asian-East, African-South, and Jewish. Most participants (88.4%, *n* = 61) practised in urban settings (100,000 people or more). Table 1 summarizes the participant demographics.

**Table 1.** Characteristics of Study Participants (*N* = 69).

Characteristics	*n* (%)
Gender	
Female	48 (69.6)
Male	19 (27.5)
Prefer not to answer	2 (3)
Two spirit	0 (0)
Gender queer	0 (0)
Other	0 (0)
Participant age	
25–34	9 (13.0)
35–44	28 (40.6)
45–54	12 (17.4)
55–64	11 (16.0)
65–75	7 (10.1)
75+	2 (2.9)
Career stage	
Early career (0–5 years)	18 (26.1)
Mid-career (5–15 years)	26 (37.7)
Senior career (15+ years)	25 (36.2)
Prefer not to answer	0 (0)
Practice location	
Large population	61 (88.4)
Medium population	4 (5.8)
Rural	3 (4.3)
Remote	1 (1.4)
Primary practice location	
Ontario	38 (55.1)
Quebec	17 (24.6)
British Columbia	10 (27.5)
Alberta	2 (3)
Newfoundland and Labrador	1 (1.4)
Manitoba	1 (1.4)

### Applicability of Palliative Psychiatry and Related Concepts

**Figure 1.**
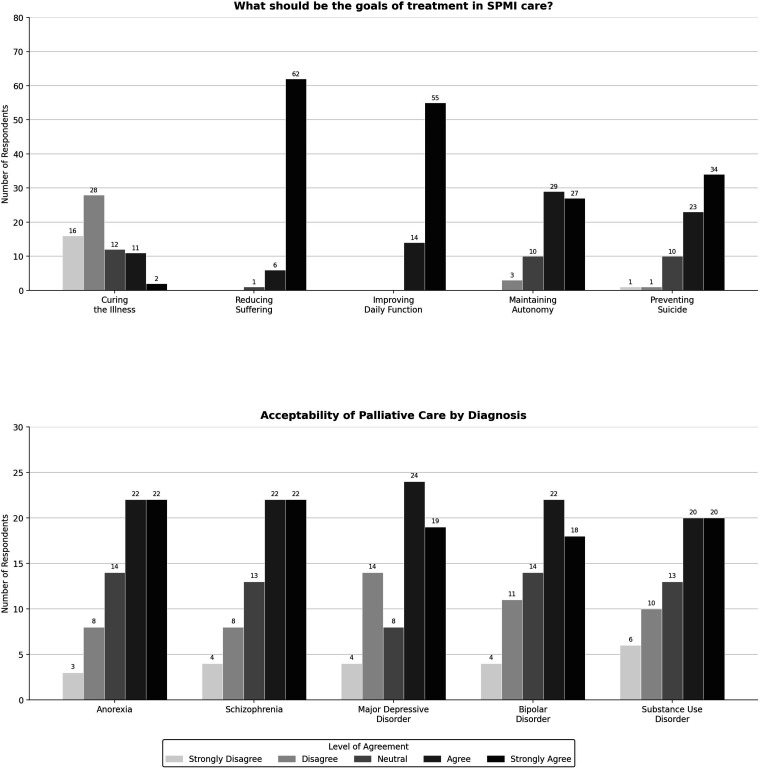
Level of agreement with different psychiatric goals of care and acceptability of palliative approaches across diagnostic categories.

Most participants agreed or strongly agreed that improving function (100%, *n* = 69), reducing suffering (98.6%, *n* = 68), preventing suicide (82.6%, *n* = 57), and maintaining patient autonomy (81.2%, *n* = 56), should be goals of treatment for people experiencing SPMI. 63.8% (*n* = 44) of participants disagreed or strongly disagreed that curing the illness should be a goal of treatment for people experiencing SPMI (Figure 1). Most participants agreed or strongly agreed that palliative care could be applied in instances of varied psychiatric diagnoses, including AN (63.8%, *n* = 44), schizophrenia (63.8%, *n* = 44), MDD (62.3%, *n* = 43), bipolar disorder (58.0%, *n* = 40), and substance use disorders (58%, *n* = 40) (Figure 1).

**Figure 2.**
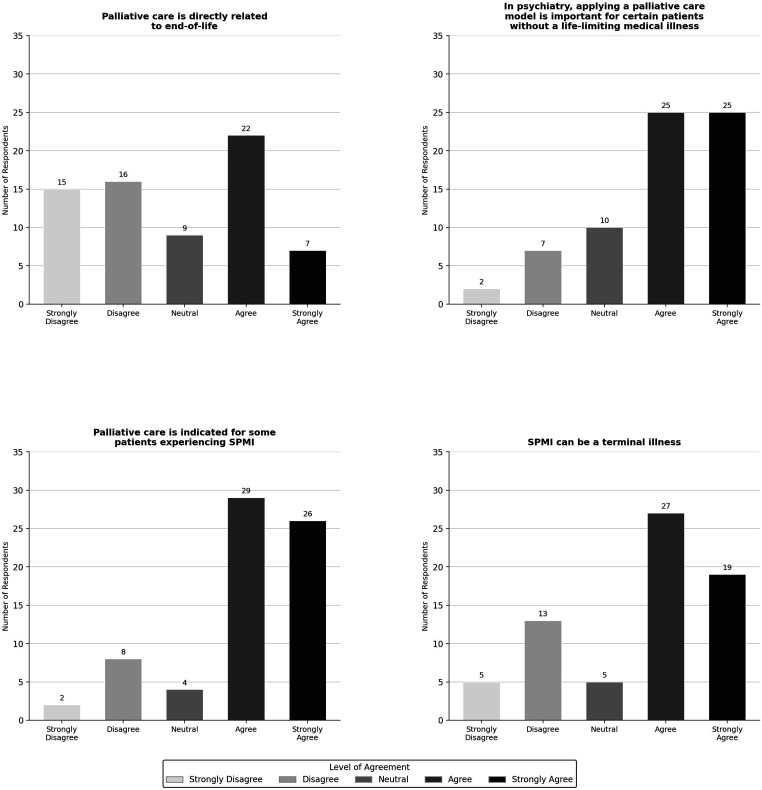
Level of agreement with statements regarding palliative care, SPMI care, and the potential relationship to concepts of terminal illness and end-of-life.

When provided with the WHO definition of palliative care^23^—which makes no mention of end-of-life care—42.0% (*n* = 29) of participants agreed or strongly agreed that palliative care was related to end-of-life, while 44.9% (*n* = 31) disagreed or strongly disagreed with that same statement (Figure 2). Most participants (72.5%, *n* = 50) agreed or strongly agreed that a palliative approach was important to providing optimal care for certain patients even without life-limiting illness (Figure 2). 79.7% (*n* = 55) of participants agreed or strongly agreed that palliative care was indicated for some patients experiencing SPMI, and 66.67% (*n* = 46) agreed or strongly agreed that SPMI could be considered a terminal illness (Figure 2).

SPMI = severe and persistent mental illness.

### Case Vignettes

#### Quantitative responses

**Figure 3.**
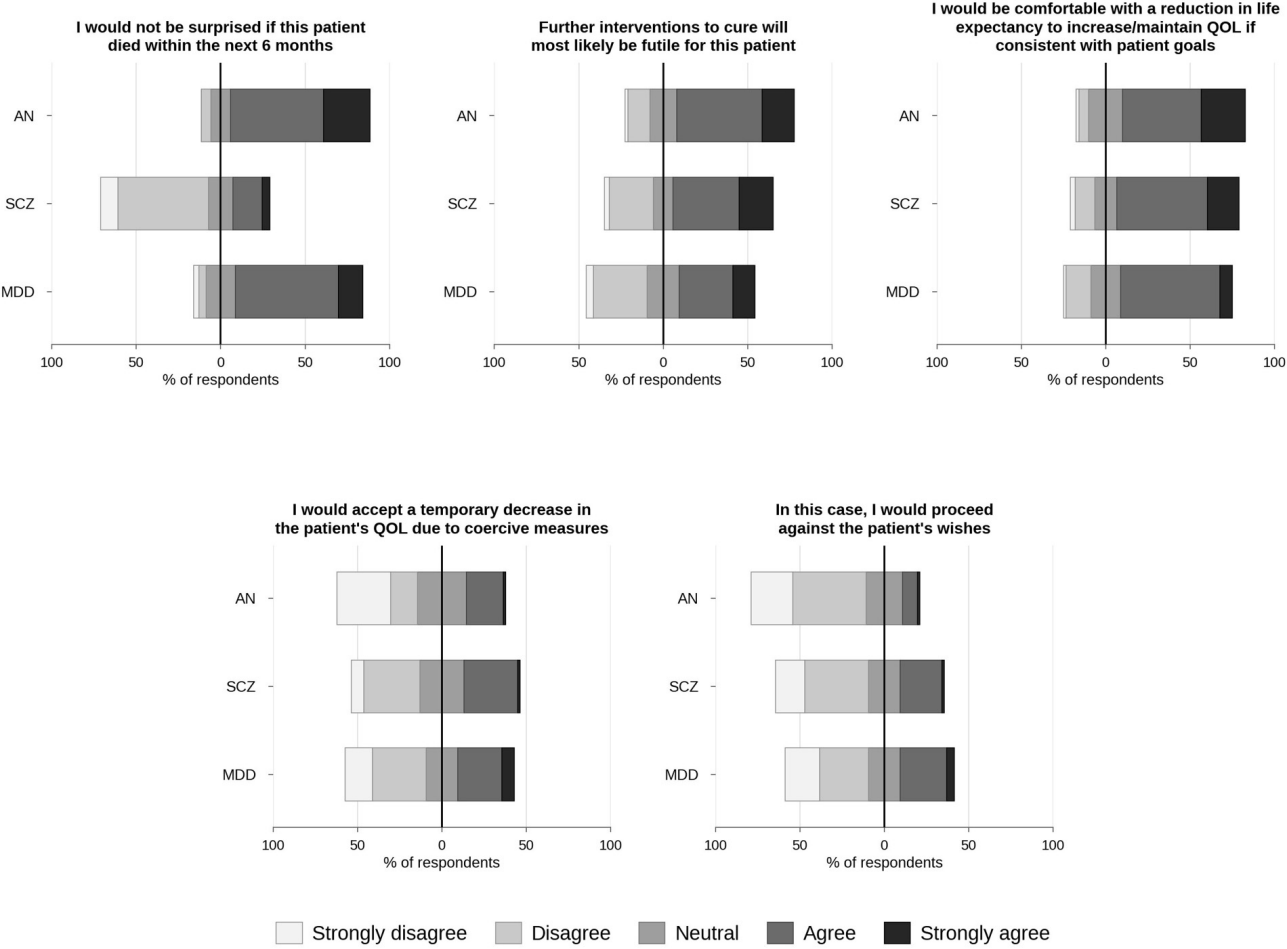
Levels of agreement regarding statements related to the 3 case vignettes (anorexia nervosa (AN), schizophrenia (SCZ), and major depressive disorder (MDD)).

Most participants agreed or strongly agreed that they would not be surprised if the person experiencing AN (82.6%, *n* = 57) or MDD (75.4%, *n* = 52) were to die in the next 6 months, but only 21.7% agreed or strongly agreed with the same statement in relation to the schizophrenia vignette (Figure 3).

Participants’ opinions about the futility of further interventions for treatment differed across diagnostic categories, with 69.6% (*n* = 48) agreeing or strongly agreeing this was the case in the instance of AN and 59.4% (*n* = 41) agreeing or strongly agreeing the same in the case of schizophrenia. The responses to the question of futility of further interventions were highly varied in the case of MDD; 44.9% (*n* = 31) agreed or strongly agreed, 18.8% (*n* = 13) were neutral, and 36.2% (*n* = 25) disagreed or strongly disagreed (Figure 3). Across cases (AN and schizophrenia = 72.5% (*n* = 50), MDD = 65.2% (*n* = 45)), most participants agreed or strongly agreed that they would be comfortable with a reduction in life expectancy to maintain QOL if consistent with patient goals (Figure 3).

Agreeing or disagreeing with futility appeared inversely correlated to accepting a decreased QOL from coercion (ρ = −0.43, 95% CI [−0.61, −0.22] in the AN vignette; ρ = −0.32, 95% CI [−0.52, −0.09] in the MDD vignette; ρ = −0.27, 95% CI [−0.47, −0.03] in the schizophrenia vignette). Responding to the AN case, 47.8% (*n* = 33) of participants disagreed or strongly disagreed with a reduction in QOL due to coercive measures, and 28.9% (*n* = 20) were neutral. For the schizophrenia case, 40.6% (*n* = 28) disagreed or strongly disagreed with this same statement, while 33.3% (*n* = 23) agreed or strongly agreed that this was acceptable. Participants were similarly split in response to the MDD case; nearly 48% (*n* = 33) disagreed or strongly disagreed with the statement and 33.3% (*n* = 23) agreed or strongly agreed with it (Figure 3). Most participants disagreed or strongly disagreed with proceeding against patient wishes in the case of AN (68.1%, *n* = 47) and schizophrenia (55.1%, *n* = 38). Participants’ responses were more divided in the case of MDD, where 49.3% (*n* = 34) disagreed or strongly disagreed with proceeding against patient wishes and 30.4% (*n* = 22) agreed or strongly agreed with this course of action (Figure 3).

#### Thematic analysis of free-text responses

Our ST-TA of participants’ free-text responses regarding the case vignette statements generated 6 themes reflecting their reasoning: ethical dimensions (59%), clinical paradigms (52%), characteristics of mental illness (35%), considering comprehensive treatment options (32%), psychosocial factors (19%), and systemic factors (18%). There were 47 associated sub-themes. The most frequent ethical dimension subthemes included capacity to consent (34%), futility (30%), and coercion (30%). The dominant subthemes under clinical paradigms included uncertainty (37%), palliative care approaches (34%), and MAiD (29%). The most common subthemes under characteristics of mental illness were harm to self and others (25%), suicide (18%), and age (14%). Access to new/experimental treatment was the primary subtheme (76%) under considering comprehensive treatment options, followed by brain stimulation (20%) and psychotherapy (20%). Family and caregivers and patient wishes (each 47%) were equally frequent subthemes under psychosocial factors, with lack of community supports (13%) as less frequent. Law (57%), social determinants of health (29%), and the Mental Health Act (14%) were the 3 most frequent subthemes under systemic factors. See Table 2 for further information about the themes, subthemes, their relation, and illustrative quotations.

**Table 2.** Results From the ST-TA Listing Theme, Subtheme, and Illustrative Quotations.

Theme	Theme Frequency (Percentage = No of Theme Codes/No of Free-Text Comments)	Subtheme	Subtheme Frequency (Percentage = No of Subtheme/No of Total Subthemes)	Illustrative Quotations
Ethical dimensions	57 (59%)	Capacity to consent	16 (21%)	“I have seen older individuals recover even with multiple failed treatments in the past, so I don’t know that treatment is ever absolutely futile. I also struggle with the effect of starvation on insight and capacity to make decisions.” (AN Vignette, Woman, Early Career) “… things can still change for her. I would not give up, even if it means just seeing her in supportive psychotherapy.” (AN Vignette, Man, Senior Career) “Our obligation is one of means, not of outcome. Multiple treatments have failed. The patient is deemed competent. We need to make her as comfortable as possible, with appropriate interventions. This is not equivalent to end-of-life care, but to a more global vision of her reality.” (AN Vignette, Woman, Mid-Career) “I realise that I have less stigma towards the depressed person, and that I tend to be more accepting of their choice to give up on life.” (MDD Vignette, Woman, Mid-Career)
		Futility	14 (19%)	
		Coercive	14 (19%)	
		Hopeful	10 (13%)	
		Risk vs. benefit	8 (11%)	
		Insight	7 (9%)	
		Stigma	2 (3%)	
		Patient abandonment	1 (1%)	
		Slippery slope	1 (1%)	
		Moral distress	1 (1%)	
		Duty of physician	1 (1%)	
Clinical paradigms	41 (52%)	Uncertainty	15 (23%)	“If the patient expressed a desire for treatment at a future point it could alter the possibility of the treatment being futile.” (AN Vignette, Woman, Early Career) “If we properly use the available tools (e.g., authorization of care orders) and if we properly adjust the treatment (e.g., Clozapine ± Abilify ± Amisulpride), this kind of situation should not occur. Out of hundreds of patients, I have had maybe 1-2 cases of this kind. Nothing to justify the widespread use of MAiD in psychiatry! And MAiD is not about “shortening the life expectancy” of a patient, but about inducing their death!” (Schizophrenia Vignette, Man, Mid-Career) “Medicolegally I am not sure how you could defend yourself if you did not take coercive action in this kind of case. I'm not sure that it's right to do so but I also don’t think we can allow people to suicide with the way our medical system is currently structured. I would be very worried about my license. This is the kind of case where MAiD might make sense.” (MDD Vignette, Woman, Early Career)
		Palliative care approaches	14 (22%)	
		MAiD	12 (18%)	
		Medication management	9 (14%)	
		Involuntary treatment	6 (9%)	
		Practice patterns	4 (6%)	
		Curative	2 (3%)	
		Recovery	1 (2%)	
		Harm reduction	1 (2%)	
		Therapeutic relationship	1 (2%)	
Characteristics of mental illness	28 (35%)	Harm to self and others	7 (19%)	“To me the key factor here is the extensive past treatment trials. I would be more in favour of coercive treatment if there had been less tried in the past. I am still curious to know what is getting in the way of recovery and if trauma is a factor and if there has been trauma treatment. I wouldn’t give up completely on a cure but it doesn’t seem imminent.” (MDD Vignette, Woman, Early Career) “He's still young, after all. Late improvements aren’t uncommon, and there's so much research underway.” (Schizophrenia Vignette, Man, Senior Career) “His young age challenges me and makes me doubt, but he has nonetheless been suffering greatly for more than 15 years.” (Schizophrenia Vignette, Woman, Senior Career)
		Suicide	5 (14%)	
		Age	4 (11%)	
		Impaired cognition	4 (11%)	
		Multiple diagnoses	3 (8%)	
		QOL	3 (8%)	
		Trauma	3 (8%)	
		Physical health	2 (6%)	
		Suffering	2 (6%)	
		Chronicity	1 (3%)	
		Treatment adherence	1 (3%)	
		Terminal illness	1 (3%)	
Considering comprehensive treatment options	25 (32%)	Access to new and/or experimental treatments	19 (56%)	“I struggle to answer this not knowing what types of psychotherapy have been provided - there are multiple, evidence, based therapies for adult anorexia - most of which are not readily accessible in Canada. I think I would feel differently if I knew she had had access to multiple, adequate, trials.” (AN Vignette, Woman, Early Career) “Newer drugs treatments can be effective, but coercion may still be hard to justify.” (Schizophrenia Vignette, Man, Senior Career) “Many new treatments can be helpful, Ketamine, cariprazine, rapid TMS, so I do not give up.” (MDD Vignette, Man, Senior Career)
		Brain stimulation	5 (15%)	
		Psychotherapy	5 (15%)	
		Assertive Community Treatment	4 (12%)	
		Multiple treatments	1 (3%)	
Psychosocial factors	15 (19%)	Family/caregiver	7 (41%)	“This is very difficult. Frankly, an important factor would be patient's family/involvement/level of acceptance of the terminal severe nature of her illness and how they are coping with her suffering.” (AN Vignette, Man, Mid-Career) “There is not a lot said about the patient's wishes here.” (Schizophrenia Vignette, Woman, Mid-Career)
		Patient wishes	7 (41%)	
		Lack of community support	2 (12%)	
		Spirituality	1 (6%)	
Systemic factors	14 (18%)	Law	8 (50%)	“Nobody working in this area expects to cure schizophrenia … this population is stigmatized and abandoned by our system enough as it is, and the idea of throwing up our hands and saying ‘well we couldn’t cure him so I guess we give up’ is deeply upsetting to me.” (Schizophrenia Vignette, Woman, Early Career) “Things like unemployement or independent housing are social measures that can be acted upon. I also feel it is a slippery slope to say that someone without friends or family (ie, without advocates) should be left with limited treatment options.” (Schizophrenia Vignette, Woman, Senior Career) “When a patient is detainable or detained under the mental health act it is much more challenging cognitively to switch to a palliative approach.” (Schizophrenia Vignette, Woman, Mid-Career)
		Social determinants of health	4 (25%)	
		Mental Health Act	2 (13%)	
		Access to resources	1 (6%)	
		Treatments available in Canada	1 (6%)	

## Discussion

We surveyed 69 Canadian psychiatrists to understand their attitudes towards using palliative care approaches in instances of SPMI. Most participants were women, White, and worked in large urban centres, though there was representation from multiple Canadian provinces. There was a wide distribution of participant ages, which may reflect varied durations of work experience in mental healthcare. A significant limitation of this work was the small sample size, despite concerted and repeated recruitment efforts. Our study is not powered to make broad claims about Canadian psychiatrists’ opinions on palliative psychiatry writ large. We situate this sample's responses within the wider literature of similar populations surveyed in other Western contexts, while expanding previous work by providing texture to our quantitative findings through a qualitative analysis of participants’ explanations of their responses where available.

Both the German-speaking Swiss and Canadian samples endorsed the importance of supporting patient function and alleviating suffering as central mental healthcare goals, above aiming to cure the illness.^13^ Both samples accepted a reduction in life expectancy in favour of increasing or maintaining QOL across the 3 case vignettes.^13^ These results suggest that some psychiatrists might support a paradigm of care consistent with palliative care principles.^25^ Notably, the goals identified by these psychiatrists correspond to those emphasized in recovery-oriented psychiatric care.^26^ The literature debates whether palliative psychiatry represents a distinct paradigm or reflects established practices in high-quality psychiatric care.^9,27^ Concerns remain that introducing palliative psychiatry as a separate pathway could unintentionally further fragment and impede access to mental healthcare.^8^ Current evidence suggests cross-cultural consistency in valuing the reduction of suffering and functional improvement as treatment goals even when curative goals are considered relevant.^14^ Future research should clarify distinctions between the goals of palliative psychiatry and other approaches to mental healthcare.

The Canadian participants, like those similarly surveyed in Switzerland,^13^ largely agreed that palliative care approaches are acceptable across a spectrum of SPMI diagnoses and important for supporting patients without a life-limiting illness. Some participants appeared to link palliative care with end-of-life care but still recognized its value for non-life-limiting illnesses. It is already well-established outside of mental healthcare that palliative care should not be limited to end-of-life care.^28,29^ Conversely, previous scholarship has suggested that conceptualizing some instances of SPMI as a terminal illness is central to applying palliative models to SPMI care.^18^ However, our survey of practising psychiatrists points to a possible practical, experienced “everyday ethic”^30^ that renders the debate over SPMI as a terminal illness less relevant to palliative psychiatry's potential use in mental healthcare. Exploring these perspectives further could help address potential hesitation to adopt palliative approaches in mental healthcare, which often stems from the stigma attached to “palliation” and its associations with death and dying and abandonment of care.^31^

Across the case vignettes, the Canadian and German-speaking Swiss samples expressed a strong level of agreement that they would be comfortable with a reduction of life expectancy to increase or maintain QOL if consistent with patient goals.^13^ Both samples largely agreed that they would not be surprised if either the AN or the MDD individual died in the next 6 months, with most disagreeing about the same for the case of schizophrenia. These comparisons suggest that perceptions of the likelihood of mortality are related to medical fact: for example, there are clear biological drivers of mortality in an AN disease course^32^ and an awareness of the epidemiology of suicide in treatment-resistant MDD.^33–35^ This question, known was the “Surprise Question,” is used as a screening tool in other medical specialties to identify people nearing end-of-life who could potentially benefit from palliative care.^36^ Future research should investigate how it can similarly be incorporated into psychiatric practice to help guide clinical decision-making.

As compared to the Canadian sample, the German-speaking Swiss sample had greater agreement that further interventions to cure the illness would be futile, especially for the schizophrenia and MDD cases.^13^ Futility is a contested term, and futility judgments are normative and are influenced by healthcare context, wider culture, personal values, and professional identity.^37,38^ Questions around how to incorporate prognostic uncertainty and interpretations of decisional capacity into futility judgments remain.^17,18^ However, our results suggest that medical facts may also be integrated into futility judgments: both Canadian and German-speaking Swiss respondents showed the highest futility agreement for the AN case, likely due to clear psychological and physiological reasons why the believed future interventions would not work,^39–41^ which are difficult to state as concretely for schizophrenia and MDD. In the Canadian sample, there was greater uncertainty (indicated by a neutral response) or disagreement about futility, especially in MDD, with qualitative findings indicating considerations of access to new and experimental treatments as a prominent subtheme among participant free-text responses. These results align with ongoing research into novel MDD therapies (e.g., psychedelics, neuroactive steroids), which has accelerated in the years between when the Swiss and Canadian studies were completed.^42–44^ The result that agreeing or disagreeing with futility was inversely correlated to accepting a decreased QOL due to coercive measures is consistent with previous qualitative findings that moral distress amongst psychiatrists is often related to the use of coercion in perceived instances of treatment futility.^45^

A strength of our study is that we qualitatively examined the reasoning behind participants’ survey responses. Analysis of participants’ elaborations on their case vignette responses revealed complex decision-making, grounded in ethics and professionalism, philosophies of care, medical facts, and both individual and systemic factors. High-frequency ethical subthemes (e.g., futility, coercion) matched the survey questions, suggesting strong participant appetite for and high level of engagement with these issues in the context of SPMI care. Participants expressed different clinical approaches (e.g., harm reduction, recovery, curative) and often referenced the challenge of managing uncertainty. Previous research demonstrates that psychiatrists accept some risk to mitigate uncertainty, provided the risk-taking is “defensible.”^46^ Our findings suggest that considering palliative approaches to mental illness can intensify the challenge of finding this balance, as legal obligations related to preventing harm to society are often weighed heavily at the systemic level. Additionally, uncertainty in psychiatric prognosis remains a central concern for palliative psychiatry.^47,48^ Future research should explore uncertainty and its management when considering potential palliation of mental illness to aid clinical decision-making.

The qualitative results suggest that our participants seek to incorporate holistic understandings of their patients when evaluating the appropriateness of palliative care approaches. For example, participants frequently cited psychosocial considerations, including individual social supports, and demonstrated an understanding of the impact of the social determinants of health. They also acknowledged the role of family, caregivers, and patient wishes when responding to the case vignettes. Principles of shared decision-making, recognized as essential to ethical palliative psychiatry^49^ and high-quality mental healthcare,^50–52^ were reflected in participant responses.

Finally, it was striking how often MAiD was mentioned in the free-text responses, despite our study abstaining from asking any questions on the topic. This suggests that discussions of palliative psychiatry in Canada may be impacted by the ongoing debate about MAiD-MI-SUMC, which has generated strong responses from across the profession.^21,53^ Canadian legislation is both contentious and unique in allowing for findings of “irremediability” when patients have capably refused previously recommended treatment.^54^ Participants often referenced legal frameworks, and we wonder how potential awareness of this legislative nuance may impact participants’ perceptions of palliative psychiatry given the close relationship between irremediability and futility.^55^ Currently, the relationship between palliative psychiatry and MAiD-MI-SUMC is poorly understood, though our group is exploring this question in more detail.^56^ Future research should explore how expanding MAiD legislation influences both psychiatrists’ and individuals experiencing SPMI's perceptions of palliative approaches to mental healthcare.

### Limitations

Limitations of this study include its small sample size, despite intensive recruitment efforts. The small sample size may reflect a self-selection bias, as those with strong opinions about palliative psychiatry (either positively or negatively) may have been more likely to participate. This limits the generalizability of our findings to the wider population of practising Canadian psychiatrists. Moreover, there was an underrepresentation of psychiatrists working outside urban centres. This study likely did not capture the opinions of psychiatrists with different practice patterns and resource availability. We do not know if we have representation of perspectives across practice types as we did not specifically ask participants about their practice focus (e.g., emergency psychiatry, community psychiatry, forensic psychiatry). This study can be viewed as an initial solicitation of certain Canadian psychiatrists’ opinions, but a future study that is more adequately powered is necessary to draw broader conclusions about the acceptability of palliative psychiatry amongst Canadian psychiatrists.

## Conclusion

Palliative psychiatry is an emerging model of mental healthcare aimed at improving QOL and mitigating suffering in instances of SPMI. Among Canadian psychiatrists surveyed there was widespread recognition of its relevance and necessity for SPMI care. Respondents endorsed the applicability of palliative approaches across diverse diagnoses, and, both generally and in vignette-based responses, affirmed the acceptability of prioritizing QOL over curative goals. Qualitative analysis revealed nuanced clinical reasoning, with participants integrating ethical principles, established and emerging clinical paradigms, individual patient factors, and sociocultural context. Future research is needed to better understand any gaps between theoretical acceptance of palliative psychiatry and practical implementation, understand cross-cultural differences in these perspectives and attitudes, and assess perspectives from additional interest-holders, including individuals with lived experience of SPMI.

## Supplemental Material

sj-docx-1-cpa-10.1177_07067437261474291 - Supplemental material for A Survey of Canadian Psychiatrists’ Attitudes Towards Palliative Care Approaches for Severe and Persistent Mental Illness: Enquête sur les attitudes des psychiatres canadiens à l’égard des approches de soins palliatifs pour les troubles mentaux graves et persistants Supplemental material, sj-docx-1-cpa-10.1177_07067437261474291 for A Survey of Canadian Psychiatrists’ Attitudes Towards Palliative Care Approaches for Severe and Persistent Mental Illness: Enquête sur les attitudes des psychiatres canadiens à l’égard des approches de soins palliatifs pour les troubles mentaux graves et persistants by Sarah Levitt, Lucy Panko, Kate Tsiandoulas, Micaela Forte, Lucy Costa and Daniel Z. Buchman in The Canadian Journal of Psychiatry

## References

[1] Zumstein N Riese F . Defining severe and persistent mental illness—a pragmatic utility concept analysis. Front Psychiatry. 2020;11:648. doi:10.3389/fpsyt.2020.00648 32733295 PMC7358610

[2] Trachsel M Irwin SA Biller-Andorno N Hoff P Riese F . Palliative psychiatry for severe and persistent mental illness. Lancet Psychiatry. 2016;3(3):200. doi:10.1016/S2215-0366(16)00005-5 26946387

[3] Westermair AL Buchman DZ Levitt S Perrar KM Trachsel M . Palliative psychiatry in a narrow and in a broad sense: a concept clarification. Aust N Z J Psychiatry. 2022;56(12):1535–1541. doi:10.1177/00048674221114784 35999690 PMC9679794

[4] Davidson L Rowe M Tondora J O’Connell MJ Lawless MS . A practical guide to recovery-oriented practice: tools for transforming mental health care. New York: Oxford University Press; 2020.

[5] Trachsel M Irwin SA Biller-Andorno N Hoff P Riese F . Palliative psychiatry for severe persistent mental illness as a new approach to psychiatry? Definition, scope, benefits, and risks. BMC Psychiatry. 2016;16:260. doi:10.1186/s12888-016-0970-y 27450328 PMC4957930

[6] Aftab A . What should clinicians know about palliative psychopharmacology? AMA J Ethics. 2023;25(9):E710–E717. doi:10.1001/amajethics.2023.710 37695874

[7] Levitt S Cooper RB Gupta M , et al. Palliative psychiatry: research, clinical, and educational priorities. Ann Palliat Med. 2024;13(3):542–557. doi:10.21037/apm-23-471 38769803

[8] Lindblad A Helgesson G Sjöstrand M . Towards a palliative care approach in psychiatry: do we need a new definition? J Med Ethics. 2019;45(1):26–30. doi:10.1136/medethics-2018-104944 30266796

[9] Strand M Sjöstrand M Lindblad A . A palliative care approach in psychiatry: clinical implications. BMC Med Ethics. 2020;21(1):29. doi:10.1186/s12910-020-00472-8 32306966 PMC7168959

[10] Gaind KS . “Palliative psychiatry” and assisted suicide: compassion? Abandonment? Or something far worse? Psychiatr Times; 2024 Nov 22. Accessed March 20, 2026. https://www.psychiatrictimes.com/view/palliative-psychiatry-and-assisted-suicide-compassion-abandonment-or-something-far-worse

[11] Braslow J . Palliative psychiatry: concerns. Psychiatr Clin North Am. 2026. In press. doi:10.1016/j.psc.2026.01.005. 42547121

[12] Decorte I Verfaillie F Moureau L , et al. Oyster Care: an innovative palliative approach towards SPMI patients. Front Psychiatry. 2020;11:509. doi:10.3389/fpsyt.2020.00509 32581883 PMC7294963

[13] Trachsel M Hodel MA Irwin SA Hoff P Biller-Andorno N Riese F . Acceptability of palliative care approaches for patients with severe and persistent mental illness: a survey of psychiatrists in Switzerland. BMC Psychiatry. 2019;19:111. doi:10.1186/s12888-019-2091-x 30975122 PMC6458682

[14] Stoll J Mathew A Venkateswaran C Prabhakaran A Westermair AL Trachsel M . Palliative psychiatry for patients with severe and persistent mental illness: a survey on the attitudes of psychiatrists in India compared to psychiatrists in Switzerland. Front Psychiatry. 2022;13:858699. doi:10.3389/fpsyt.2022.858699 35693967 PMC9178077

[15] Gloeckler S Trachsel M . Nurses’ views on palliative care for those diagnosed with severe persistent mental illness: a pilot survey study in Switzerland. J Psychiatr Ment Health Nurs. 2022;29(1):67–74. doi:10.1111/jpm.12742 33580631

[16] van Veen SMP Ruissen AM Widdershoven GAM . Irremediable psychiatric suffering in the context of physician-assisted death: a scoping review of arguments. Can J Psychiatry. 2020;65(9):613–620. doi:10.1177/0706743720923072 PMC7457463 32427501

[17] Geppert CMA . Futility in chronic anorexia nervosa: a concept whose time has not yet come. Am J Bioeth. 2015;15(7):34–43. doi:10.1080/15265161.2015.1039720 26147264

[18] Levitt S Buchman DZ . Applying futility in psychiatry: a concept whose time has come. J Med Ethics. 2021;47(12):e60. doi:10.1136/medethics-2020-106672 33443107

[19] Verdegem S Rens A Vandenberghe J , et al. Relating to life and death: a qualitative study of individuals with a long-lasting death wish related to unbearable psychiatric suffering. Int J Qual Stud Health Well-Being. 2025;20(1):2469361. doi:10.1080/17482631.2025.2469361 40009633 PMC11866764

[20] Vanhie T Verdegem S Cuypers A Bemelmans L Onghena P Vandenberghe J . The Reakiro care model for persons with a chronic death wish: an epidemiological study. J Humanist Psychol. 2025;65(1):1–22. doi:10.1177/00221678241313088

[21] Dembo J Schuklenk U Reggler J . “For their own good”: a response to popular arguments against permitting medical assistance in dying (MAID) where mental illness is the sole underlying condition. Can J Psychiatry. 2018;63(7):451–456. (14). doi:10.1177/0706743718766055 29635929 PMC6099778

[22] Canadian Psychiatric Association. Medical assistance in dying (MAiD): CPA position statement. Ottawa (ON): Canadian Psychiatric Association; 2021. Accessed March 20, 2026. https://www.cpa-apc.org/wp-content/uploads/2021-CPA-Position-Statement-MAID-Update-EN-web-Final.pdf (15)

[23] World Health Organization. Palliative care. Geneva: World Health Organization; 2020 Aug 5. Accessed March 20, 2026. https://www.who.int/news-room/fact-sheets/detail/palliative-care

[24] Robinson OC . Conducting thematic analysis on brief texts: the structured tabular approach. Qual Psychol. 2022;9(2):194–208. doi:10.1037/qup0000189

[25] Woods A Willison K Kington C Gavin A . Palliative care for people with severe persistent mental illness: a review of the literature. Can J Psychiatry. 2008;53(11):725–736. doi:10.1177/070674370805301104 19087466

[26] Barber ME . Recovery as the new medical model for psychiatry. Psychiatr Serv. 2012;63(3):277–279. doi:10.1176/appi.ps.201100248 22388534

[27] Kious BM Nelson RH . Does it matter whether a psychiatric intervention is “palliative"? AMA J Ethics. 2023;25(9):E655–E660. doi:10.1001/amajethics.2023.655 37695866

[28] Hawley PH . The bow tie model of 21st century palliative care. J Pain Symptom Manage. 2014;47(1):e2–e5. doi:10.1016/j.jpainsymman.2013.10.009 24321509

[29] Sawatzky R Porterfield P Lee J , et al. Conceptual foundations of a palliative approach: a knowledge synthesis. BMC Palliat Care. 2016;15:5. doi:10.1186/s12904-016-0076-9 26772180 PMC4715271

[30] Zizzo N Bell E Racine E . What is everyday ethics? A review and a proposal for an integrative concept. J Clin Ethics. 2016;27(2):117–128. doi:10.1086/JCE2016272117 27333062

[31] Alcalde J Zimmermann C . Stigma about palliative care: origins and solutions. Ecancermedicalscience. 2022;16:1377. doi:10.3332/ecancer.2022.1377 35702413 PMC9116991

[32] Mehler PS Watters A Joiner T Krantz MJ . What accounts for the high mortality of anorexia nervosa? Int J Eat Disord. 2022;55(5):633–636. doi:10.1002/eat.23664 34997783

[33] Reutfors J Andersson TM-L Brenner P , et al. Mortality in treatment-resistant unipolar depression: a register-based cohort study in Sweden. J Affect Disord. 2018;238:674–679. doi:10.1016/j.jad.2018.06.030 29966932

[34] Bergfeld IO Mantione M Figee M Schuurman PR Lok A Denys D . Treatment-resistant depression and suicidality. J Affect Disord. 2018;235:362–367. doi:10.1016/j.jad.2018.04.016 29665520

[35] Rost F Booker T Gonsard A , et al. The complexity of treatment-resistant depression: a data-driven approach. J Affect Disord. 2024;358:292–301. doi:10.1016/j.jad.2024.04.093 38697222

[36] White N Kupeli N Vickerstaff V Stone P . How accurate is the ‘surprise question’ at identifying patients at the end of life? A systematic review and meta-analysis. BMC Med. 2017;139. doi:10.1186/s12916-017-0907-4 PMC5540432 28764757

[37] Westermair AL Trachsel M . Moral intuitions about futility as prompts for evaluating goals in mental health care. AMA J Ethics. 2023;25(9):E690–E702. doi:10.1001/amajethics.2023.690 37695872

[38] Kious BM Levitt S van Veen SMP , et al. Addressing ‘futility’ in psychiatry: a consensus statement. Psychol Med. 2026;56:e16. doi:10.1017/S0033291725102961 PMC12885334 41531198

[39] Walsh BT . The enigmatic persistence of anorexia nervosa. Am J Psychiatry. 2013;170(5):477–484. doi:10.1176/appi.ajp.2012.12081074 23429750 PMC4095887

[40] Frank GKW . From desire to dread—a neurocircuitry based model for food avoidance in anorexia nervosa. J Clin Med. 2021;10(11):2228. doi:10.3390/jcm10112228 34063884 PMC8196668

[41] Frank GKW Shott ME Stoddard J Swindle S Pryor TL . Association of brain reward response with body mass index and ventral striatal-hypothalamic circuitry among young women with eating disorders. JAMA Psychiatry. 2021;78(10):1123–1133. doi:10.1001/jamapsychiatry.2021.1580 34190963 PMC8246338

[42] Goodwin GM Aaronson ST Alvarez O , et al. Single-dose psilocybin for a treatment-resistant episode of major depression. N Engl J Med. 2022;387(18):1637–1648. doi:10.1056/NEJMoa2206443 36322843

[43] Winslow M White E Rose SJ Salzer E Nemec EC . The efficacy of zuranolone versus placebo in postpartum depression and major depressive disorder: a systematic review and meta-analysis. Int J Clin Pharm. 2024;46(3):586–594. doi:10.1007/s11096-024-01714-0 38489051

[44] Njenga C Ramanuj PP de Magalhães FJC Pincus HA . New and emerging treatments for major depressive disorder. Br Med J. 2024;386:e073823. doi:10.1136/bmj-2022-073823 38977279

[45] Austin WJ Kagan L Rankel M Bergum V . The balancing act: psychiatrists’ experience of moral distress. Med Health Care Philos. 2008;11(1):89–97. doi:10.1007/s11019-007-9083-1 17703374

[46] Dixon M Oyebode F . Uncertainty and risk assessment. Adv Psychiatr Treat. 2007;13(1):70–78. doi:10.1192/apt.bp.105.001297

[47] Berk M Berk L Castle D . When illness does not get better: do we need a palliative psychiatry? Acta Neuropsychiatr. 2008;20(5):272–274. doi:10.1111/j.1601-5215.2008.00316.x

[48] Trauer T . Commentary on: palliative models of care for later stages of mental disorder: maximising recovery, maintaining hope and building morale. Aust N Z J Psychiatry. 2012;46(2):170–172. doi:10.1177/0004867411435287 22311533

[49] Brown VA Trust A . What makes palliative mental health care ethical health care? AMA J Ethics. 2023;25(9):E674–E677. doi:10.1001/amajethics.2023.674 37695869

[50] Adams JR Drake RE Wolford GL . Shared decision-making preferences of people with severe mental illness. Psychiatr Serv. 2007;58(9):1219–1221. doi:10.1176/ps.2007.58.9.1219 17766569

[51] Matthias MS Salyers MP Rollins AL Frankel RM . Decision making in recovery-oriented mental health care. Psychiatr Rehabil J. 2012;35(4):305–314. doi:10.2975/35.4.2012.305.314 22491370 PMC3980461

[52] Huang C Plummer V Lam L Cross W . Perceptions of shared decision-making in severe mental illness: an integrative review. J Psychiatr Ment Health Nurs. 2020;27(2):103–127. doi:10.1111/jpm.12558 31444919

[53] Downie J . Ending toxicity: a call to reintroduce constructive discourse about contentious health-related issues in medicine. Can J Bioeth. 2025;8(1-2):159–161. doi:10.7202/1117882ar

[54] Kirby J . Interpreting irremediability when a mental health disorder is the sole-qualifying medical condition for MAiD. Can J Bioeth. 2022;5(4):83–88. doi:10.7202/1094700ar

[55] Van Veen SMP Evans N Ruissen AM Vandenberghe J Beekman ATF Widdershoven GAM . Irremediable psychiatric suffering in the context of medical assistance in dying: a Delphi-study. Can J Psychiatry. 2022;67(10):758–767. doi:10.1177/07067437221087052 35311599 PMC9510999

[56] Forte M Panko L Tsiandoulas K , et al. The relationship between palliative psychiatry and medical assistance in dying for mental illness: a registered scoping review. OSF; October 7 2025. Accessed March 20, 2026. https://osf.io/fr6a8/overview

