# Supplementary material for: A Survey of Canadian Psychiatrists’ Attitudes Towards Palliative Care Approaches for Severe and Persistent Mental Illness: Enquête sur les attitudes des psychiatres canadiens à l’égard des approches de soins palliatifs pour les troubles mentaux graves et persistants
Source: Can J Psychiatry. 2026 Aug 12:07067437261474291. Online ahead of print. doi: 10.1177/07067437261474291 (PMC13473329; doi:10.1177/07067437261474291)
Supplement: Supplementary file 1 [file sj-docx-1-cpa-10.1177_07067437261474291.docx]

**Appendix 1**. *Complete Survey Questions Examining Canadian Psychiatrists’ Attitudes Toward Palliative Psychiatry*

1. Demographic Questions: In order to better understand who is participating in the study we are collecting relevant demographic information.
   a. Please identify your gender(s)
      i. Woman
      ii. Man
      iii. Two Spirit *(An umbrella term encompassing gender and sexual diversity in Indigenous communities. Two Spirit people often serve integral and important roles in their communities, such as leaders and healers. There are many understandings the term Two Spirit – and this English term does not resonate for everyone. Two-Spirit is a cultural term reserved for those who identify as Indigenous).*
      iv. Genderqueer/Gender non-conforming/Non-binary *(A person whose gender identity is neither man nor woman, is between or beyond genders, or is some combination of genders).*
      v. Other
      vi. Do not know
      vii. Prefer not to answer
   b. Please select your age range
      i. 18-24
      ii. 25-34
      iii. 35-44
      iv. 45-54
      v. 55-64
      vi. 65-74
      vii. 75 or older
      viii. Prefer not to answer
   c. Which of the following best describes how you identify? Please select all that apply.
      i. African – North (e.g. origins include Canadian, Moroccan, or Algerian)
      ii. African – South (e.g. origins include Canadian, South African, Zimbabwean)
      iii. African – East (e.g., origins include Canadian, Ethiopian, and Somali)
      iv. African – West (e.g., origins include Canadian, Senegal, Guinea, Cote d’Ivoire)
      v. Asian- Central (e.g., origins include Canadian or Afghan)
      vi. Asian-East (e.g. Canadian, Chinese, Japanese, Korean)
      vii. Asian-Southeast (e.g., origins include Canadian, Malaysian, Filipina/o/x, Vietnamese, Thai)
      viii. Asian-South (e.g. origins include Canadian, Indian, Pakistani, Sri Lankan)
      ix. Black-African (e.g., origins include Canadian, Ghanaian, Kenyan, Somali)
      x. Black-North American (e.g., origins include Canadian, American)
      xi. Black-Caribbean (e.g., origins include Canadian, Barbadian, Jamaican)
      xii. First Nations
      xiii. Indian-Caribbean (e.g., origins include Canadian, Guyanese, with origins in India)
      xiv. Indigenous/Aboriginal
      xv. Inuit
      xvi. Latin American (e.g., origins include Canadian, Argentinian, Chilean, El Salvadorian)
      xvii. Metis
      xviii. Middle Eastern (e.g., origins include Canadian, Egyptian, Iranian, Lebanese)
      xix. White-European (e.g., origins include Canadian, English, Italian, Portuguese, Russian)
      xx. White North American (e.g., origins include Canadian, American)
      xxi. Mixed Heritage (e.g., origins include Canadian, Black-African and White-North American)
      xxii. Prefer not to answer
      xxiii. Do not know
      xxiv. Group not identified above.
            1. Specify:
   d. Please select your career stage:
      i. Early Career (0-5 years)
      ii. Mid-Career (5-15 years)
      iii. Senior Career (15 years or more)
      iv. Prefer not to answer
   e. What Province or Territory is the location of your primary practice (if more than one)? Please select one.
      i. Alberta
      ii. British Columbia
      iii. Manitoba
      iv. New Brunswick
      v. Newfoundland and Labrador
      vi. Northwest Territories
      vii. Nova Scotia
      viii. Nunavut
      ix. Ontario
      x. Prince Edward Island
      xi. Saskatchewan
      xii. Quebec
      xiii. Yukon
      xiv. Prefer not to answer
   f. How would you describe your primary practice location
      i. Large Urban Population Centre (100,000 people or over)
      ii. Medium Population Centre (30,000- 99,999 people)
      iii. Small Population Centre (1,000- 29,999 people)
      iv. Rural (less than 1,000 people)
      v. Remote (at least 350km from the nearest service centre that has year-round road access)
      vi. Prefer not to answer
      vii. Don’t know
1. Questions on the Treatment of Patients with Severe and Persistent Mental Illness (SPMI). Please indicate your level of agreement regarding the goals of treatment when working with patients experiencing severe and persistent mental illness (SPMI):
   a. Curing the illness
      i. *Strongly disagree, disagree, neutral, agree, strongly agree.*
   b. Reducing suffering
      i. *Strongly disagree, disagree, neutral, agree, strongly agree.*
   c. The patient’s ability to function in daily life
      i. *Strongly disagree, disagree, neutral, agree, strongly agree.*
   d. The patient remaining autonomous
      i. *Strongly disagree, disagree, neutral, agree, strongly agree.*
   e. Preventing suicide
      i. *Strongly disagree, disagree, neutral, agree, strongly agree.*
2. According to the World Health Organization (WHO), Palliative Care “is an approach that improves the quality of life of patients and their families facing the problem associated with life-threatening illness, through the prevention and relief of suffering by means of early identification and impeccable assessment and treatment of pain and other problems, physical, psychosocial, and spiritual.”

*Please enter your level of agreement with the following:*

a. For me, the term “palliative” is directly related to end-of-life.
   i. *Strongly disagree, disagree, neutral, agree, strongly agree.*
b. For some patients experiencing SPMI, palliative care is indicated.
   i. *Strongly disagree, disagree, neutral, agree, strongly agree.*
c. In psychiatry, applying a palliative care model is important to provide optimal support for certain patients without a life-limiting medical illness.
   i. *Strongly disagree, disagree, neutral, agree, strongly agree.*
d. In some cases of severe, enduring and treatment-refractory anorexia nervosa, a palliative care approach would be suitable.
   i. *Strongly disagree, disagree, neutral, agree, strongly agree.*
e. In some cases of severe, chronic and treatment-refractory schizophrenia, a palliative care approach would be suitable.
   i. *Strongly disagree, disagree, neutral, agree, strongly agree.*
f. In some cases of severe, chronic and treatment-refractory major depressive disorder, a palliative care approach would be suitable.
   i. *Strongly disagree, disagree, neutral, agree, strongly agree.*
g. In some cases of severe, chronic and treatment-refractory bipolar disorder, a palliative care approach would be suitable.
   i. *Strongly disagree, disagree, neutral, agree, strongly agree.*
h. In some cases of severe, chronic and treatment-refractory substance use disorder, a palliative care approach would be suitable.
   i. *Strongly disagree, disagree, neutral, agree, strongly agree.*
i. SPMI can be a terminal illness.
   i. *Strongly disagree, disagree, neutral, agree, strongly agree.*
j. Continuous palliative sedation in a patient for the reduction of unbearable refractory psychological symptoms is justifiable in certain cases of SPMI.
   i. *Strongly disagree, disagree, neutral, agree, strongly agree.*

**Case Vignettes**

In order to improve our understanding of whether palliative care is considered acceptable in different situations, please provide an evaluation of the case vignettes below.

1. Patient 1: 37-year-old woman Anorexia Nervosa, onset at age 11. Symptoms: general muscle weakness, loss in bone density, amenorrhea. Current weight 24kg / 52 lbs., BMI 9.5 kg/m2, no recent weight gain or stabilization, no acute danger of dying as her body is adapted to being underweight. The patient went through 10 previous inpatient treatments (in both somatic and psychiatric hospitals), of which 3 took place in specialized psychiatric institutions. Different approaches of intensive psychotherapy throughout the course of disease have been tried, without success. During her hospitalizations, the patient went through several artificial re-feedings, sometimes under sedation. The patient now refuses artificial re-feeding and treatment. She states that for years her life has only focused on having to overcome her anorexia, leaving her with neither friends nor hobbies. She suffers from the physical symptoms, including general muscle weakness and loss in bone density. She voices that she would rather die than undergo further treatment and wishes to be left in peace. She does not want to be forced into eating anymore. The patient is capable to consent.

*Please enter your level of agreement with the following:*

a. I would not be surprised if this patient died within the next 6 months.
   i. *Strongly disagree, disagree, neutral, agree, strongly agree.*
b. For this patient, further interventions to cure the anorexia will most likely be futile.
   i. *Strongly disagree, disagree, neutral, agree, strongly agree.*
c. In this case, I would be comfortable with a reduction of life expectancy to increase or maintain quality of life if consistent with the patient’s goals.
   i. *Strongly disagree, disagree, neutral, agree, strongly agree.*
d. In this case, I would accept a temporary decrease in the patient’s quality of life due to coercive measures.
   i. *Strongly disagree, disagree, neutral, agree, strongly agree.*
e. In this case, I would proceed against the patient’s wishes
   i. *Strongly disagree, disagree, neutral, agree, strongly agree.*
f. Please provide any reflections you have on the case in relation to your responses:

2. Patient 2: 33-year-old man, Schizophrenia with onset at age 17, no significant comorbidities. Positive symptoms: auditory and visual hallucinations, persecutory delusions. Negative symptoms: apathy, social withdrawal, poverty of speech (all rated severe). Despite long-lasting high-dose pharmacological treatment (several atypical neuroleptics, haloperidol, clozapine and their combinations, augmentation therapies) as well as electro-convulsive therapy, the patient has never been free from positive symptoms. Multiple psychotherapies with various approaches have also failed to stabilize the patient or improve his quality of life. He does not want to continue assertive community treatment because he feels it is too intrusive. While the positive symptoms were more dominant in the first years after his initial diagnosis, he developed severe negative symptoms as well as aggression and self-injurious behavior such as burning himself with cigarettes. The negative symptoms and his strong functional deficits are exacerbated by chronic unemployment and the inability to live independently. Furthermore, the patient has no family system. His persisting illness has left him completely isolated with no social contacts and no hobbies or interests. He recognizes that his symptoms are related to a mental disorder. The patient is capable to consent.

*Please enter your level of agreement with the following:*

a. I would not be surprised if this patient died within the next 6 months.
   i. *Strongly disagree, disagree, neutral, agree, strongly agree.*
b. For this patient, further interventions to cure the schizophrenia will most likely be futile.
   i. *Strongly disagree, disagree, neutral, agree, strongly agree.*
c. In this case, I would be comfortable with a reduction of life expectancy to increase or maintain quality of life if consistent with the patient’s goals.
   i. *Strongly disagree, disagree, neutral, agree, strongly agree.*
d. In this case, I would accept a temporary decrease in the patient’s quality of life due to coercive measures.
   i. *Strongly disagree, disagree, neutral, agree, strongly agree.*
e. In this case, I would proceed against the patient’s wishes.
   i. *Strongly disagree, disagree, neutral, agree, strongly agree.*
f. Please provide any reflections you have on the case in relation to your responses:

3. Patient 3: 40-year-old man. Recurrent Major Depressive Disorder without significant comorbidities. Somatic symptoms: Energy loss, insomnia, and fatigue. Persistent suicidal ideation over the past 20 years, current acute and concrete suicidal intent. The patient went through intensive evidence-based long-term psychotherapy of different approaches, including specialized treatment approaches such as CBT and IPT. Neither solely psychotherapeutic nor the combination with adequate treatment trials of antidepressants (selective serotonin reuptake inhibitors, tricyclic antidepressants, venlafaxine, augmentation with lithium and antipsychotic medications (quetiapine and aripiprazole)) led to the improvement of his depression. The patient experienced significant adverse effects with several of the medications. Exhausted and as a last resort, he has decided to undergo electroconvulsive therapy. However, maintenance electroconvulsive therapy was equally insufficient to prevent recurrence of suicidal ideation. The symptoms even worsened. The patient experiences severe hopelessness and states that his quality of life is very poor, "that he doesn't want to deal with his illness anymore," and that he plans to attempt suicide in the near future. He continues to be capable to consent to treatment.

*Please enter your level of agreement with the following:*

a. I would not be surprised if this patient died within the next 6 months
   i. *Strongly disagree, disagree, neutral, agree, strongly agree.*
b. For this patient, further interventions to cure the MDD will most likely be futile.
   i. *Strongly disagree, disagree, neutral, agree, strongly agree.*
c. In this case, I would be comfortable with a reduction of life expectancy to increase or maintain quality of life if consistent with the patient’s goals.
   i. *Strongly disagree, disagree, neutral, agree, strongly agree.*
d. In this case, I would accept a temporary decrease in the patient’s quality of life due to coercive measures.
   i. *Strongly disagree, disagree, neutral, agree, strongly agree.*
e. In this case, I would proceed against the patient’s wishes.
   i. *Strongly disagree, disagree, neutral, agree, strongly agree.*
f. Please provide any reflections you have on the case in relation to your responses:

Thank you very much for completing this survey!
